# Dynamics of Extracellular Traps in Vaginal Dysbiosis Associated with *Gardnerella vaginalis*: *Ex Vivo* Evidence from Neutrophils and Monocytes

**DOI:** 10.3390/ijms27135932

**Published:** 2026-07-01

**Authors:** Aurora Prado-Sanhueza, Angélica Melo, Isabel Iturrieta-González, Pablo Navarro, Fabiola Zambrano

**Affiliations:** 1 Doctoral Program in Morphological Sciences, Universidad de La Frontera, Temuco 4811230, Chile; a.prado01@ufromail.cl; 2 Center of Excellence in Translational Medicine-Scientific and Technological Bioresource, Faculty of Medicine, Universidad de La Frontera, Temuco 4780000, Chile; angelica.melo@ufrontera.cl (A.M.); isabel.iturrieta@ufrontera.cl (I.I.-G.); 3 Department of Preclinical Sciences, Faculty of Medicine, Universidad de La Frontera, Temuco 4780000, Chile; 4 Faculty of Health Sciences, Universidad Autónoma de Chile, Temuco 4811230, Chile; pablo.navarro@ufrontera.cl

**Keywords:** neutrophil extracellular traps (NETs), macrophage extracellular traps (METs), bacterial vaginosis, *Gardnerella vaginalis*, vaginal dysbiosis, ex vivo imaging

## Abstract

Vaginal dysbiosis, particularly bacterial vaginosis (BV), is associated with altered immune responses that may influence the formation of extracellular traps (ETs). This study aimed to characterize neutrophil and macrophage extracellular traps (NETs and METs) in vaginal discharge samples obtained from women with normal microbiota (NM) and BV, with particular emphasis on *Gardnerella vaginalis* (GV) detection. An *ex vivo* analysis was performed using vaginal smears from 14 patients previously classified according to the Nugent criteria. Immunofluorescence assays targeting neutrophil elastase (NE), citrullinated histone H3 (Citr-H3), CD15, and CD68 were conducted, and quantitative image analysis was performed using the TissueFAXS and StrataQuest platforms. NETs were classified into three morphotypes: spread (*spr*), diffuse (*diff*), and aggregated (*agg*). BV samples exhibited a substantially higher mean NET count than NM samples (842.43 vs. 91.86). The number of *diff*NETs was significantly higher in BV samples than in NM samples (*p* = 0.004), whereas GV-positive samples showed increased *spr*NET abundance compared with that in negative samples (248 vs. 8; *p* < 0.05). CD68^+^ cell counts were significantly higher in BV samples (*p* = 0.026), whereas no significant differences in NE or Citr-H3 fluorescence intensity were observed between groups. MET structures were also identified, suggesting macrophage involvement in the local immune response. Collectively, these findings indicate that vaginal dysbiosis and GV presence are associated with enhanced NET formation and distinct morphotype distributions, supporting a role for ETs in the immunopathology of BV.

## 1. Introduction

Vaginal infections represent a major global public health concern because of their well-established association with adverse reproductive outcomes, including pregnancy loss, preterm birth, low birth weight, infertility, and increased susceptibility to sexually transmitted infections, such as human immunodeficiency virus, human papillomavirus, and *Chlamydia trachomatis*. These complications are largely driven by exacerbated inflammatory responses within the vaginal microenvironment [1,2]. Among these conditions, bacterial vaginosis (BV) is one of the most prevalent polymicrobial infections affecting women of reproductive age and continues to impose a substantial global burden. According to the World Health Organization (2023), BV affects approximately 30% of women worldwide, with reported prevalence estimates ranging from 23% to 29% [3,4].

BV is not a conventional single-pathogen infection but rather the consequence of progressive vaginal microbiota dysbiosis. Under physiological conditions, the vaginal niche is dominated by *Lactobacillus* spp., which may constitute up to 86.7% of the microbial community. In contrast, *Gardnerella vaginalis* (GV), although detectable at low abundance (approximately 7.2%) in healthy microbiota, is consistently recognized as a key contributor to BV pathogenesis [5]. As dysbiosis progresses, the vaginal ecosystem shifts toward an intermediate microbiota characterized by a marked increase in GV (up to 23%) and the expansion of other anaerobic species, such as *Megasphaera* spp. and *Atopobium vaginae*. This transition is accompanied by a pronounced depletion of *Lactobacillus* spp. (down to 7.1%), ultimately leading to the establishment of BV [5]. Despite this well-described microbial transition, the host immune mechanisms that shape, amplify, or fail to resolve this dysbiotic state remain incompletely understood.

Among BV-associated microorganisms, GV was selected as the primary focus of this study because it is considered a key initiator of the polymicrobial biofilm characteristic of BV. As one of the earliest colonizers of the vaginal epithelium, GV facilitates the establishment of other BV-associated bacteria and produces virulence factors, including vaginolysin and sialidases, that promote epithelial disruption, immune activation, and microbial persistence. Therefore, GV represents a biologically relevant model for investigating extracellular trap (ET) responses during vaginal dysbiosis [6,7].

Innate immune cells, particularly polymorphonuclear neutrophils and macrophages, play a central role in the first line of defense within the vaginal mucosa. Both cell types are capable of releasing ETs, which are web-like structures composed of decondensed chromatin decorated with antimicrobial proteins. In neutrophils, these structures are referred to as neutrophil extracellular traps (NETs), whereas in macrophages they are termed macrophage extracellular traps (METs). ETs contribute to host defense by capturing and neutralizing a broad spectrum of pathogens, including bacteria and fungi through extracellular mechanisms. Notably, alterations in the vaginal microenvironment, such as those induced by bacterial, fungal, or parasitic infections, represent potent stimuli that promote ET formation, particularly through the induction of neutrophil ETosis (NETosis) [8,9]. Although NETs are rapidly released by neutrophils and primarily contribute to acute antimicrobial defense, METs play additional roles in pathogen containment, immune regulation, and tissue remodeling within mucosal environments [10].

Although ETs are traditionally regarded as antimicrobial defense mechanisms, accumulating evidence suggests that excessive or persistent ET formation may also contribute to BV pathogenesis. ETs can promote local inflammation through the release of histones, proteases, and reactive oxygen species (ROS), potentially compromising epithelial barrier integrity and disrupting mucosal homeostasis. Furthermore, interactions between ETs and polymicrobial biofilms may influence bacterial persistence and the chronic inflammatory milieu characteristic of BV [11,12].

Structurally, approximately 70% of NETs consist of histone-bound DNA, along with a diverse array of antimicrobial proteins derived from neutrophil granules, including neutrophil elastase (NE), myeloperoxidase, and cathepsin G [13,14]. Mechanistically, ET formation is triggered by the activation of NADPH oxidase (NOX), leading to a burst of ROS [15,16]. This oxidative signal promotes the translocation of peptidyl arginine deiminase type IV (PAD4) in neutrophils [17] and PAD2 in macrophages [18,19], enzymes that catalyze histone citrullination and drive chromatin decondensation. The process culminates in nuclear expansion, disruption of the nuclear and plasma membranes, and the release of chromatin fibers into the extracellular space, thereby forming NETs or METs. Although these mechanisms have been extensively characterized in systemic inflammatory contexts, their specific dynamics and regulation within the *vaginal milieu*, particularly during dysbiosis, remain insufficiently explored.

Recent *in vivo* studies have highlighted the relevance of ETs in the vaginal environment. Recently was described distinct ET phenotypes in vaginal fluids from patients with different microbiota states and infections, including *Candida albicans*, *Trichomonas vaginalis*, and BV. These phenotypes were categorized as diffuse (“*diff*”), spread (“*spr*”), and aggregated (“*agg*”) structures, reflecting morphological heterogeneity that may be functionally relevant [12]. More recently, was reported that NET formation is a common feature across infectious and non-infectious vaginal inflammatory conditions, including BV, candidiasis, trichomoniasis, and cytolytic vaginosis [20]. Notably, ETs were also detected in the absence of identifiable pathogens, suggesting that these structures may play a dual role by either contributing to microbial control or exacerbating local inflammation [20]. Unlike soluble inflammatory mediators, ET morphotypes provide insight into host–pathogen interactions by reflecting distinct antimicrobial and immunomodulatory responses within the mucosal microenvironment [12].

We hypothesized that BV, particularly in the presence of GV, is associated with increased ET formation and altered morphotype distribution within the vaginal microenvironment. Therefore, the aim of this observational *ex vivo* study was to characterize NETs and METs in vaginal fluid samples obtained from women with normal microbiota (NM) and BV and to evaluate their association with GV presence.

## 2. Results

### 2.1. Detection of GV by PCR

**Figure 1:**
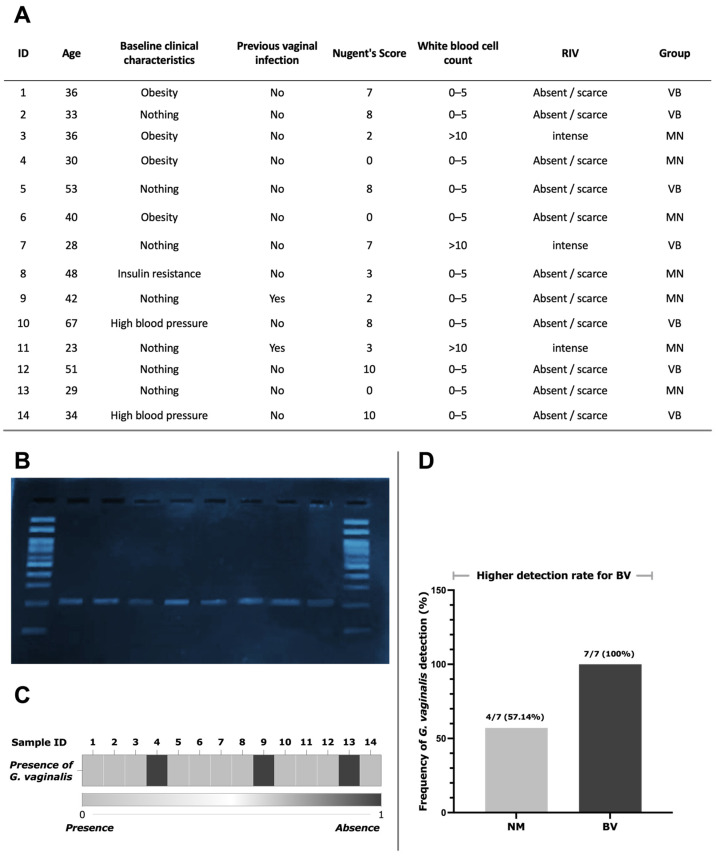
Detection of *Gardnerella vaginalis* (GV) by PCR in clinical samples. (**A**) Classification and characterization of clinical samples, including age, comorbidities, previous vaginal infections, Nugent score, white blood cell count, and classification of the vaginal inflammatory response (RIV). (**B**) Representative agarose gel electrophoresis showing PCR amplification of *G. vaginalis* from individual samples. Bands at the expected amplicon size indicating positive detection. (**C**) Frequency of GV detection according to microbiological status. Although the difference was not statistically significant (*p* = 0.096), GV was detected in 4/7 NM samples (57.14%) and 7/7 BV samples (100%), indicating a higher prevalence in BV-associated microbiota. (**D**) Comparative frequency of GV detection in samples from women with normal microbiota (NM) and bacterial vaginosis (BV).

Gram staining confirmed the previously established diagnoses of the archived samples, which included seven NM and seven BV samples. Of the 14 samples analyzed, 11 yielded amplification products consistent with the expected size of the GV-specific amplicon (206 bp). Of these, seven corresponded to BV samples and four to NM samples. The remaining three samples, all belonging to the NM group, tested negative for GV. No nonspecific bands or evidence of cross-contamination were observed in the agarose gels. Subsequently, the PCR results for GV detection were organized into a binary heat map and a frequency map based on microbiological status; however, the differences were not statistically significant according to Fisher’s exact test (*p* = 0.096) (Figure 1).

### 2.2. Quantification of CD15^+^ and CD68^+^ Cells in NM and BV Samples by Immunofluorescence (IF)

**Figure 2:**
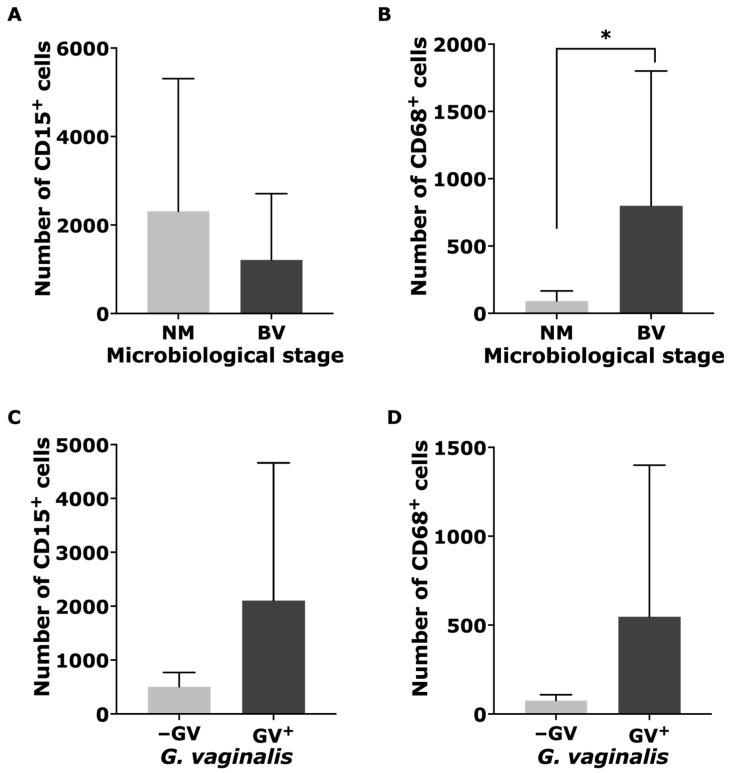
Differential distribution of innate immune cell markers across experimental groups. (**A**) CD15^+^ neutrophil counts stratified by microbiological status. (**B**) CD68^+^ monocyte/macrophage counts according to microbiological status. (**C**) CD15^+^ neutrophil counts based on the presence or absence of GV. (**D**) CD68^+^ monocyte/macrophage counts based on the presence or absence of GV. Data are presented as mean ± standard deviation (SD), and differences were considered statistically significant at *p* < 0.05.

IF analysis revealed that, across all evaluated plates, CD15^+^ cell counts ranged from 115 to 7067, corresponding to percentages ranging from 2.83% to 54.51%, with a mean of 1208.6 cells per plate. In the NM group, CD15^+^ cell counts ranged from 134 to 7067, with percentages ranging from 10.11% to 54.51%. In contrast, the BV group exhibited CD15^+^ cell counts ranging from 115 to 3496 and percentages from 2.83% to 50.96%. No statistically significant differences in neutrophil counts were observed between the groups (*p* = 0.383) (Figure 2A).

For CD68^+^ cells, the total count ranged from 17 to 2815, with a mean of 799.4 cells. In the NM group, CD68^+^ cell counts ranged from 17 to 221, whereas in the BV group they ranged from 84 to 2815. In this case, a statistically significant difference was observed between the groups (*p* = 0.026) (Figure 2B).

Among the GV-positive plates (n = 11), CD15^+^ cell counts ranged from 115 to 7067, with a mean of 2103.3 cells, whereas CD68^+^ cell counts ranged from 17 to 2815, with a mean of 546.8 cells. No statistically significant differences for either marker were observed in this analysis (Figure 2C,D).

### 2.3. Distribution of NETotic and METotic Cells by Microbiological Status and GV Presence

**Figure 3:**
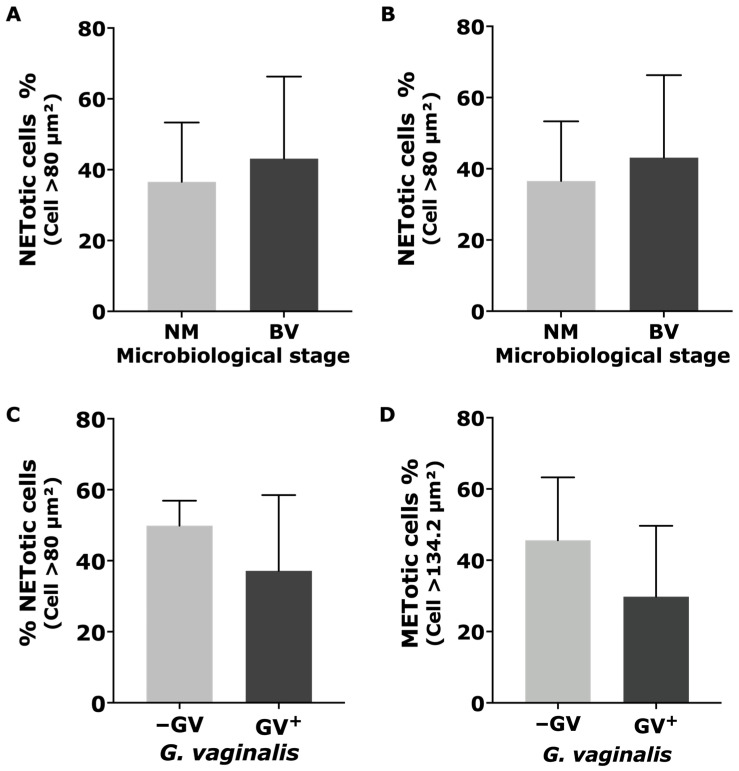
Distribution of NETotic and METotic cells by microbiological status and GV presence. (**A**) Percentage of NETotic cells (CD15^+^) according to microbiological status (NM vs. BV). (**B**) Percentage of METotic cells (CD68^+^) according to microbiological status (NM vs. BV). (**C**) Percentage of NETotic cells (CD15^+^) in relation to the presence or absence of GV. (**D**) Percentage of METotic cells (CD68^+^) in relation to the presence or absence of GV. Data are presented as mean ± SD, and differences were considered significant at *p* < 0.05.

The proportion of NETotic cells across all analyzed plates ranged from 11.38% to 76.52%. When stratified by microbiological status, the NM group exhibited values ranging from 11.94% to 54.03%, whereas the BV group showed a broader distribution, ranging from 11.38% to 76.52% (Figure 3A). The total proportion of METotic cells ranged from 2.01% to 55.92%. Specifically, the NM group exhibited values ranging from 3.17% to 44.74%, whereas the BV group displayed a wider range, from 2.01% to 55.92% (Figure 3B).

Among the GV-positive plates (n = 11), the proportion of NETotic cells within the CD15^+^ population ranged from 11.38% to 76.52%. Similarly, the proportion of METotic cells within the CD68^+^ population ranged from 2.01% to 55.92% (Figure 3C,D). Overall, both NETosis and macrophage ETosis (METosis) exhibited substantial variability across samples, with a tendency toward broader distributions in BV-associated conditions, suggesting heterogeneous activation of ET formation in response to microbiota composition and GV presence.

### 2.4. Quantitative and Morphological Characterization of ETs by NE and Citrullinated Histone H3 (Citr-H3) IF

**Figure 4:**
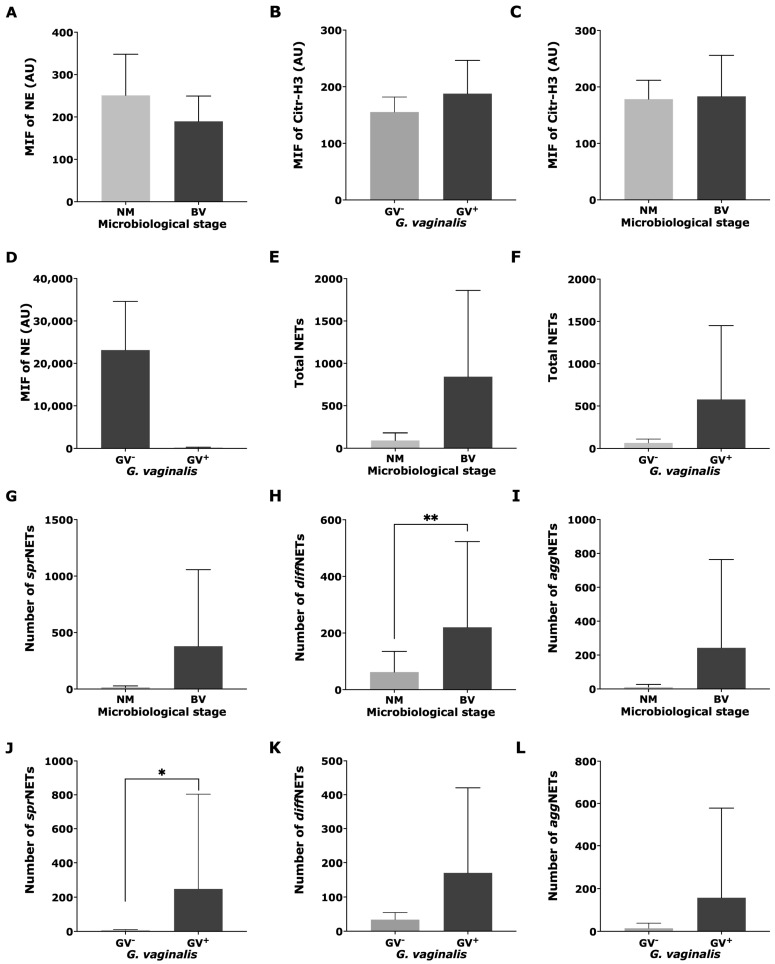
Immunofluorescence (IF) intensity and quantitative distribution of NETs and their morphotypes according to microbiological status and GV presence. (**A**,**B**) Mean Florescence Intensity (MFI) of neutrophil elastase (NE) and citrullinated histone H3 (Citr-H3) in NM and BV samples. (**C**,**D**) IF intensity of NE and Citr-H3 stratified by the presence (GV^+^) or absence (–GV) of GV. (**E**) Total NET counts according to microbiological status (NM vs. BV). (**F**) Total NET count according to GV presence (GV^+^ vs. –GV). (**G**–**I**) Quantification of NET morphotypes—spread (*spr*NETs), diffuse (*diff*NETs), and aggregated (*agg*NETs)—comparing NM and BV samples with a predominance of *spr*NETs in BV samples. A statistically significant increase in *diff*NETs was observed in BV samples compared with that in NM samples (*p* < 0.01) (**H**). (**J**–**L**) Distribution of NET morphotypes based on GV detection. A significant increase in *spr*NETs was observed in GV^+^ samples compared with those in –GV samples (*p* < 0.05) (**J**), whereas *diff*NETs (**K**) and *agg*NETs (**L**) showed no statistically significant differences. Data are presented as mean ± SD. Statistical significance is indicated as * *p* < 0.05 and ** *p* < 0.01.

IF analysis targeting NE and Citr-H3 revealed comparable ranges of fluorescence intensity between microbiological conditions, with greater variability observed in BV. Specifically, NE fluorescence intensity ranged from 133.03 to 408.37 AU in NM samples and from 131.56 to 304.38 AU in BV samples. For Citr-H3, fluorescence intensity ranged from 135.73 to 227.02 AU in NM samples and from 131.50 to 333.10 AU in BV samples (Figure 4A–D).

A marked increase in total NET formation was observed in BV samples compared with that in NM samples. Across all samples, total NET counts ranged from 18 to 2520, with an overall mean of 467.14. Stratified analysis showed a substantially higher mean in BV (842.43) than that in NM (91.86). Morphotype-specific evaluation revealed that *spr*NETs were the predominant structures in BV samples (379.29), followed by *agg*NETs (242.86) and *diff*NETs (220.29). In contrast, NM samples were characterized by a predominance of *diff*NETs (62.29), with lower representation of *spr*NETs (14.43) and *agg*NETs (10.86). A statistically significant increase was observed specifically for *diff*NETs in BV compared with those in NM samples (*p* = 0.004; Figure 4H), whereas differences in *spr*NETs (*p* = 0.259) and *agg*NETs (*p* = 0.209) did not reach statistical significance.

Consistent with these findings, the distribution of NET morphotypes highlighted a shift in structural dominance associated with microbiological status, with *spr*NETs predominating in BV samples and *diff*NETs in NM samples (Figure 4E–I). Representative IF images further confirmed the presence and morphological diversity of these structures (Figure 4A–C).

When stratifying by the presence of GV, positive samples exhibited a clear increase in NET formation. The mean total NET count in GV-positive plates was 577, compared with 66 in negative samples (all of which corresponded to NM samples). Morphotype analysis showed higher mean counts in positive samples (*spr*NETs: 248; *diff*NETs: 171; *agg*NETs: 158) than those in negative samples (*spr*NETs: 8; *diff*NETs: 34; *agg*NETs: 14). This difference was statistically significant for total NET counts (Figure 4J), supporting a strong association between GV detection and enhanced NETosis.

### 2.5. IF Characterization of ET-like Structures and Associated Cellular Markers

**Figure 5:**
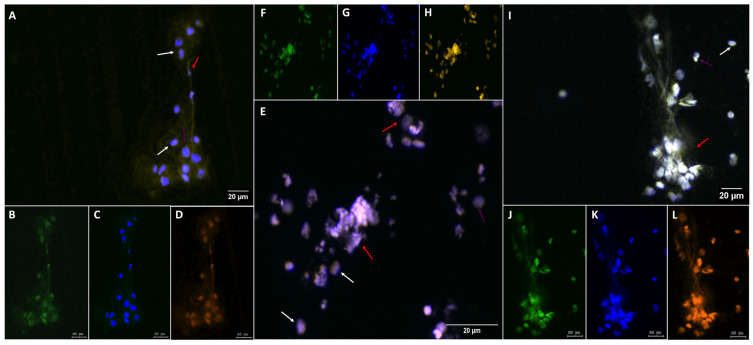
IF characterization of extracellular DNA structures and associated cellular markers. Representative images showing the distribution and organization of extracellular DNA and cellular components. (**A**) Merged fluorescence image displaying the co-localization of DNA (DAPI; blue) with cellular markers. (**B**–**D**) Individual fluorescence images illustrating the spatial distribution of each marker. (**E**) Higher-magnification image showing compact DNA aggregates surrounded by dispersed cells. (**F**–**H**) Individual fluorescence images highlighting the partial co-localization and spatial segregation of fluorescence signals. (**I**) Elongated filamentous structures consistent with extracellular DNA release, with aligned nuclei and diffuse chromatin signals. (**J**–**L**) Individual fluorescence images corresponding to panel (**I**), confirming the distribution of each marker within the observed structures. The arrows indicate representative regions of interest; the white arrows indicate epithelial cells, while the red arrows indicate extracellular traps and the purple arrows indicate neutrophils. Scale bars: 20 μm.

IF analysis revealed the presence of extracellular DNA structures associated with inflammatory cells across the evaluated samples. Elongated and clustered DNA-positive structures (DAPI-stained) co-localizing with cellular markers and forming dense aggregates and filamentous extensions suggestive of ET-like formations were observed (Figure 5). Analysis of merged fluorescence channels showed the spatial organization of these structures, in which DNA signals were closely associated with cellular components (Figure 5A). Individual fluorescence channels highlighted the distribution of specific markers and showed distinct yet overlapping patterns (Figure 5B–D). Higher-magnification analysis revealed compact DNA aggregates surrounded by dispersed signals, indicating heterogeneous structural organization (Figure 5E). Partial co-localization and spatial segregation of fluorescence signals supported the presence of complex extracellular assemblies (Figure 5F–H). Elongated filamentous structures were also observed, with DNA signals aligned along these formations, suggesting the release and spreading of chromatin into the extracellular space (Figure 5I). The co-distribution of the evaluated markers within these structures reinforced the association between cellular components and extracellular DNA (Figure 5J–L). Overall, these findings support the presence of extracellular DNA-associated structures with variable morphologies, ranging from compact aggregates to elongated filaments, consistent with ET-like formations.

### 2.6. IF Identification of METs

**Figure 6:**
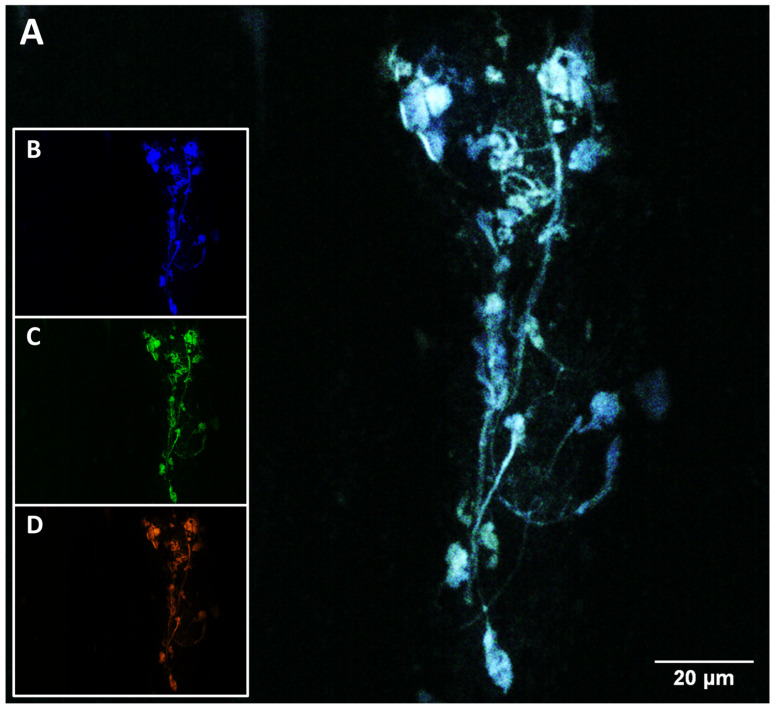
IF identification of macrophage extracellular traps (METs). (**A**) Merged fluorescence image showing extracellular DNA structures associated with CD68^+^ cells. DNA is visualized with DAPI (blue), macrophages are labeled with CD68 (green), and extracellular DNA derived from membrane-compromised cells is detected with SYTOX Orange (orange). Extensive filamentous and web-like structures are observed, extending beyond cellular boundaries. (**B**) DAPI fluorescence channel highlighting decondensed and extracellular DNA fibers. (**C**) CD68 fluorescence channel showing the distribution of macrophage-associated signals along these structures. (**D**) SYTOX Orange fluorescence channel confirming the extracellular localization of DNA. Co-localization of CD68 with extracellular DNA supports identification of these structures as METs. Scale bars: 20 μm.

IF analysis revealed the presence of extracellular DNA structures associated with CD68^+^ cells, consistent with METs. Analysis of merged fluorescence channels revealed extensive filamentous and web-like structures, characterized by diffuse and elongated DNA staining (DAPI-stained) extending beyond the cellular boundaries (Figure 6A). These extracellular DNA fibers form interconnected networks containing attached cellular remnants.

Channel-specific fluorescence images showed that CD68^+^ signals co-localized with these extracellular structures, indicating their macrophage origin (Figure 6C). SYTOX Orange staining (orange), which selectively labels extracellular DNA derived from membrane-compromised cells, overlapped with the DAPI-positive filaments, confirming the extracellular nature of the chromatin (Figure 6D). The DAPI channel alone highlighted the transition from intact nuclear morphology to decondensed and dispersed DNA structures (Figure 6B).

Morphologically, METs appeared as elongated and branching filaments, with regions of increased density forming compact aggregates interconnected by thinner strands. These structures extended across the field, suggesting active chromatin release and spatial expansion within the microenvironment. The co-distribution of CD68^+^ signals with extracellular DNA supported the identification of these structures as METs rather than passive cellular debris.

## 3. Discussion

This study provides ex vivo evidence that BV, particularly in the presence of GV, is associated with a distinct ET response characterized by enhanced NET formation and specific alterations in NET morphotype distribution. Our findings expand upon previous observations describing ETs in vaginal inflammatory conditions and provide novel evidence linking BV-associated dysbiosis with both NET- and MET-associated responses [12,21]. Importantly, the increased number of CD68^+^ cells in BV samples suggests an active involvement of macrophages in the dysbiotic vaginal microenvironment. Macrophage recruitment is a recognized feature of chronic mucosal inflammation and may contribute not only to pathogen recognition but also to the amplification of local inflammatory signaling through cytokine release and MET formation [10,22]. In addition, identification of MET-like structures in BV samples further supports a direct role for macrophages in extracellular chromatin release during vaginal dysbiosis.

Although CD68^+^ cells were significantly increased in BV samples, the relative abundance of METotic cells was lower and more variable than that of the NET response. This finding may reflect the distinct biology of macrophages, which are generally longer-lived cells specialized in phagocytosis, antigen presentation, cytokine production, and tissue repair. Consequently, MET release may represent a later or more restricted response than the rapid ET formation typically observed in neutrophils. Within the BV microenvironment, the lower relative abundance of METs may also suggest that macrophages remain viable to sustain local immune regulation and clearance functions rather than undergo extensive chromatin release. Alternatively, BV-associated bacteria, including GV, may partially evade macrophage extracellular responses through biofilm formation, sialidase activity, and other immune-modulating mechanisms that promote persistence within the vaginal niche. Therefore, the comparatively lower MET signal observed in this study may reflect both macrophage survival-focused functions and potential bacterial immune evasion strategies, which together could contribute to the chronic and recurrent nature of BV [23,24].

A major finding of this study was the significant increase in *diff*NETs in BV samples compared with that in NM samples. *diff*NETs are characterized by highly decondensed and dispersed chromatin fibers and have been associated with pronounced inflammatory activation and rapid extracellular antimicrobial responses [25]. Their predominance in BV likely reflects an enhanced innate immune response to the dysbiotic microbial community. BV-associated bacteria, including GV and other anaerobes, produce virulence factors such as vaginolysin, sialidases, and short-chain fatty acids, which are capable of activating ROS-dependent signaling pathways involved in NETosis [26,27]. Consequently, excessive *diff*NET release may perpetuate inflammation within the vaginal mucosa. Similar mechanisms have been described in other mucosal inflammatory diseases, in which persistent NET release promotes epithelial damage, oxidative stress, and immune dysregulation [11]. From a mechanistic perspective, the predominance of *diff*NETs may be linked to sustained activation of pattern-recognition receptors by the polymicrobial communities characteristic of BV. Recognition of bacterial ligands through Toll-like receptors can promote ROS generation, PAD4 activation, histone citrullination, and extensive chromatin decondensation, ultimately favoring the formation of highly dispersed NET structures. In addition, BV-associated metabolites and virulence factors may prolong neutrophil activation, thereby creating a persistent inflammatory environment that supports continued NET release. Therefore, predominance of *diff*NETs may represent not only an antimicrobial response but also a marker of dysregulated mucosal inflammation associated with vaginal dysbiosis [11].

Notably, although BV samples displayed higher total NET counts than those in NM samples, only *diff*NETs reached statistical significance, suggesting that specific NET morphotypes may represent distinct stages or functional adaptations of the innate immune response. The morphological heterogeneity of NETs has been increasingly recognized as biologically relevant, with different NET structures potentially exhibiting distinct antimicrobial and immunomodulatory properties [13,28]. *Diff*NETs may facilitate the rapid diffusion of antimicrobial proteins and chromatin across the mucosal surface, whereas more compact structures could participate in microbial sequestration [29].

An additional key observation was the significant increase in *spr*NETs in samples positive for GV. *spr*NETs are elongated and filamentous structures with an expanded surface area that may enhance bacterial trapping efficiency [5]. This finding is particularly relevant given the well-established biofilm-forming capacity of GV. Biofilm formation represents a major virulence mechanism in BV, promoting bacterial persistence, antibiotic tolerance, and evasion of host immune responses [30]. Previous studies have demonstrated that NETs can interact directly with bacterial biofilms, either limiting bacterial dissemination or, paradoxically, contributing to chronic inflammation and microbial persistence [31]. The predominance of *spr*NETs in GV-positive samples suggests that the host actively attempts to contain biofilm-associated bacteria within the vaginal lumen. Moreover, this predominance may also reflect an adaptive response to biofilm-associated growth. Unlike planktonic bacteria, biofilms form spatially organized microbial communities embedded within an extracellular matrix that restricts phagocyte access. Under these conditions, elongated and widely distributed NET structures may increase the surface area available for pathogen containment and local accumulation of antimicrobial molecules. However, persistent exposure to biofilm-derived products may simultaneously sustain chronic NET production, potentially contributing to tissue damage and maintenance of a pro-inflammatory microenvironment [17].

Notably, NE and Citr-H3 fluorescence intensities did not differ significantly between groups despite the marked differences in NET abundance and morphology. These findings suggest that ET-associated inflammatory responses in BV may be more closely related to structural remodeling and NET morphotype distribution than to overall expression levels of canonical NET markers. Similar dissociations between NET abundance and marker expression have been reported in other inflammatory conditions [17].

Taken together, our findings suggest that ET formation in BV is not merely a nonspecific inflammatory event but rather a highly dynamic and structurally regulated innate immune response associated with vaginal dysbiosis and the presence of GV. The concomitant increase in CD68^+^ cells, enhanced *diff*NET formation in BV samples, and predominance of *spr*NETs in GV-positive samples support the hypothesis that distinct ET morphotypes may arise in response to specific microbial and inflammatory cues within the vaginal microenvironment. We hypothesize that *diff*NET structures may reflect an exacerbated inflammatory state associated with mucosal instability, whereas elongated *spr*NETs may represent a more coordinated response aimed at containing biofilm-associated bacteria. However, persistent ET release may paradoxically promote chronic inflammation, epithelial damage, and microbial persistence, thereby contributing to the recurrent and often refractory nature of BV.

Overall, this study expands the current understanding of innate immune responses in BV by demonstrating that ET formation involves the coordinated participation of both neutrophils and macrophages within the vaginal microenvironment. The differential predominance of NET morphotypes according to microbiological status and GV presence suggests that ET architecture may reflect specific host–microbe interactions during vaginal dysbiosis. These observations indicate that ETs not only function as antimicrobial structures but also active modulators of mucosal inflammation and tissue homeostasis. Consequently, dysregulated ET formation may represent a key mechanistic link among microbial imbalance, persistence of inflammation, and impaired vaginal mucosal integrity in BV.

## 4. Materials and Methods

### 4.1. Study Population

A total of 70 women between 18 and 70 years of age were recruited during gynecological consultations at primary healthcare centers in Temuco, Chile. Prior to sample collection, all participants provided written informed consent. The study protocol was approved by the Scientific Ethics Committee of Universidad de La Frontera (Approval No. 045-2017).

### 4.2. Inclusion and Exclusion Criteria and Sample Selection and Processing

The inclusion criteria were women over 18 years of age who presented with abnormal vaginal discharge, as well as asymptomatic women attending gynecological appointments. The exclusion criteria were menstruation at the time of sample collection, sexual activity within the previous 48 h, antibiotic treatment within the preceding 30 d, and a history of immunodeficiency or the use of immunosuppressive therapy. Women were included if they met all study criteria and were in the follicular phase of the ovarian cycle at the time of sample collection.

For each participant, two glass slides containing vaginal fluid smears were prepared at the time of sample collection and maintained at room temperature until processing. Additionally, an aliquot of vaginal fluid was stored in isopropanol at −80 °C for subsequent DNA extraction. Separate slides were used for Gram staining and IF analyses.

This study was designed as an exploratory observational ex vivo analysis. Therefore, no a priori sample size calculation was performed. The sample size was determined based on the availability of archived clinical specimens that fulfilled all inclusion criteria, including confirmed Nugent classification and the absence of co-infections with *Candida* spp. and *T*. *vaginalis*.

### 4.3. Gram Staining and Assessment of Microbiological Status

Vaginal fluid smears were Gram-stained (Chemix, Sunnyvale, CA, USA; Cat. No. 151005) according to standard procedures. Briefly, samples were stained with crystal violet for 1 min, rinsed with distilled water, and treated with Lugol’s iodine for 1 min and rinsed again with distilled water. Slides were then decolorized with alcohol–acetone, washed, and counterstained with safranin for 1 min. After a final wash, slides were air-dried and mounted with coverslips.

Microscopic evaluation was performed under oil immersion at 100× magnification. Ten non-adjacent fields per slide were analyzed, and bacterial morphotypes were quantified, including Gram-positive bacilli (*Lactobacillus* spp.), pleomorphic Gram-variable coccobacilli (suggestive of GV), and Gram-variable curved bacilli (*Mobiluncus* spp.). Microbiological status was determined using the Nugent scoring system, in which scores of 0–3 indicate NM, whereas 7–10 are consistent with BV.

### 4.4. DNA Extraction and PCR Amplification

DNA was recovered from the frozen vaginal fluid samples by centrifugation at 12,000 rpm for 15 min at 4 °C, followed by washing with 70% ethanol and resuspension in TE buffer (10 mmol/L Tris-HCl, 1 mmol/L EDTA; pH 8).

DNA integrity and the absence of PCR inhibitors were assessed by conventional PCR targeting the β-globin gene (268 bp fragment), using the PCO4 (5′-caacttcatccacgttcacc-3′) and GH20 (5′-gaagagccaaggacaggacaggacaggtac-3′) primers [32].

GV was detected by conventional PCR using specific primers targeting a 206 bp fragment (5′-gcgggctagagtgca-3′ and 5′-acccgtggaatgggcc-3′) [33]. PCR products were resolved on 2% agarose gels (Cleaver Scientific, Rugby, UK) stained with SafeView Plus (abm^®^ Richmond, BC, Canada, Cat. No. FER00SV500UL), using a 100 bp DNA ladder (New England Biolabs, Ipswich, MA, USA) as a molecular size marker, and visualized on a UV transilluminator (Thermo Fisher Scientific, Pleasanton, CA, USA).

### 4.5. IF and ET Analyses

IF staining was performed to identify inflammatory cells and ETs in vaginal fluid smears. Neutrophils and macrophages were detected using anti-human CD15 and CD68 antibodies, respectively, whereas ETs were characterized using NE and Citr-H3 immunofluorescence.

Slides were delimited with a hydrophobic barrier 24 h prior to processing, fixed with 4% paraformaldehyde (Thermo Fisher Scientific; Cat. No. FB002) for 15 min, washed with PBS (Thermo Fisher Scientific; Cat. No. 003002), and blocked with 2% BSA (Sigma Life Science, USA; Cat. No. A331150G) in PBS for 30 min at room temperature. Negative controls were prepared by omitting the primary antibodies and replacing them with antibody diluent. No specific fluorescence signal was detected in these controls.

For cell identification, samples were incubated for 1 h in the dark with CD15-FITC (BioLegend, San Diego, CA, USA, Cat. No. 301904) and CD68-FITC monoclonal antibodies (BioLegend, San Diego, CA, USA Cat. No. 333806), followed by PBS washes and nuclear staining with Sytox Orange^®^ (1:2000; Thermo Fisher Scientific; Waltham, MA, USA, Cat. No. S11368). Slides were mounted using Fluoromount with DAPI (Thermo Fisher Scientific, Waltham, MA, USA; Cat. No. 00-4959-52) and stored protected from light for 24 h. Quantification of CD15^+^ and CD68^+^ cells was performed using StrataQuest V7.0 software (ANID FONDEQUIP EQM200228) by applying automated cell masking.

Nuclear segmentation using Sytox Orange^®^ and DAPI (Thermo Fisher Scientific; Waltham, MA, USA) enabled the morphological discrimination of ETotic cells from epithelial cells. NETosis was defined by a nuclear area >80 µm^2^ and loss of lobulation [15], whereas METosis was defined by a nuclear area >134.2 µm^2^, based on the mean macrophage nuclear area measured in NM samples (n = 7).

For ET marker detection, samples were incubated with anti-NE (1:300; Abcam; Waltham, MA, USA, Cat. No. ab68672) or anti-Citr-H3 (1:1000; Cell Signaling; Danvers, MA, USA, Cat. No. 97272S) for 2 h, followed by incubation with an Alexa Fluor™ 488-conjugated secondary antibody for 1 h (Invitrogen; Cat. No. A11008). After washing, slides were stained with Sytox Orange^®^ (1:2000; Thermo Fisher Scientific; Waltham, MA, USA).

Fluorescence imaging was performed using the TissueFAXS i Plus system (TissueGnostics, Vienna, Austria), with defined areas of 30 mm^2^ scanned for each sample. Images were analyzed using TissueFAXS-Viewer Version 7.0 and StrataQuest V7.0 software, enabling the quantification of fluorescence intensity and detection of NE- and Citr-H3-associated ETs. NET morphotypes (*spr*NETs, *diff*NETs, and *agg*NETs) were quantified across the scanned area on a field-by-field basis.

Furthermore, the origin and nature of the clinical samples present inherent analytical challenges, as vaginal secretions constitute highly heterogeneous and viscous biological matrices that may interfere with automated image segmentation and quantitative ET analysis, particularly in regions with high cell density and mucus content. Therefore, all IF staining, image acquisition, and quantitative analyses were performed using standardized protocols by the same trained operator to minimize inter-operator variability and ensure reproducibility.

### 4.6. Statistical Analysis

Data were recorded in a Microsoft Office Excel spreadsheet. Descriptive analyses were performed, and the mean and corresponding standard deviation were calculated. Data normality was assessed using the D’Agostino–Pearson K^2^ test. Comparisons between two groups were performed using the nonparametric Mann–Whitney U test, whereas comparisons involving three or more groups were conducted using the nonparametric Kruskal–Wallis test, followed by a Bonferroni-adjusted post hoc test when statistically significant differences were detected. Fisher’s exact test was used to evaluate frequency distributions among categorical variables. Statistical analyses were performed using IBM SPSS Statistics for Windows, Version 23.0 (IBM Corp., Armonk, NY, USA) and GraphPad Prism for Macintosh, Version 11.0.0 (GraphPad Software LLC, San Diego, CA, USA). Graphs were generated using GraphPad Prism. A *p*-value < 0.05 was considered statistically significant.

### 4.7. Study Limitations

Although the use of archived clinical samples enabled standardized microbiological classification, it also limited the sample size, which may have contributed to the observed data dispersion. Therefore, future studies including larger cohorts are warranted to increase statistical power and improve the generalizability of the findings. This study could be complemented by an evaluation of ET dynamics before and after standard treatment for BV, allowing for the identification of ETs as potential biomarkers. Such analyses would allow correlations between ET morphotype distribution and therapeutic response, thereby facilitating the identification of patients at higher risk of recurrence and guiding the development of individualized treatment strategies.

## Data Availability

The data supporting the findings of this study are available from the corresponding author upon reasonable request. Data are not publicly available due to privacy and ethical restrictions.

## Abbreviations

The following abbreviations are used in this manuscript:

BV	Bacterial vaginosis
NM	Normal microbiota
ETs	Extracellular traps
NETs	Neutrophil extracellular traps
METs	Macrophage extracellular traps
NE	Neutrophil elastase
Citr-H3	Citrullinated histone H3
PMN	Polymorphonuclear neutrophils
ROS	Reactive oxygen species
NOX	NADPH oxidase
PAD2	Peptidyl arginine deiminase type 2
PAD4	Peptidyl arginine deiminase type 4
PCR	Polymerase chain reaction
DAPI	4′,6-diamidino-2-phenylindole
IF	Immunofluorescence
GV	*Gardnerella vaginalis*/*G. vaginalis*
*spr*NETs	Spread neutrophil extracellular traps
*diff*NETs	Diffuse neutrophil extracellular traps
*agg*NETs	Aggregated neutrophil extracellular traps
